# Supplementary material for: Putative Mechanisms Responsible for the Antihyperglycemic Action of Lactobacillus paracasei HII01 in Experimental Type 2 Diabetic Rats
Source: Nutrients. 2020 Oct 1;12(10):3015. doi: 10.3390/nu12103015 (PMC7601916; doi:10.3390/nu12103015)
Supplement: Supplementary file 1 [file nutrients-12-03015-s001.pdf]

Supplementary Files

Table S1. The composition of normal and high-fat diet.

Ingredients	Normal diet		High-fat diet	
	g	%E	g	%E
Carbohydrates	56.21	60.11	26.38	22.54
Fat	4.55	10.95	27.89	53.63
Protein	27.06	28.94	28.81	23.93
Vitamin and mineral	6.54	-	9.92	-
Fiber	3.43	-	4.32	-
Total	97.79	100	97.32	100
kcal/g	3.74 kcal/g		4.68 kcal/g	

Energy per gram (kcal/g): Carbohydrates=4; fat=9; protein=4

Wilkes, J.J.; Boden, A.; Bell, A.; Bell, R.C. A modified high-fat diet induces insulin resistance in rat skeletal muscle but not adipocytes. Am J Physiol 1998, 275, 679-686.

Table S2. Group-specific primers of bacterial targets based on 16 S rDNA sequences.

Organisms	Forward Primer	Reverse Primer
Lactobacillus group	5′-AGCAGTAGGGAATCTTCCA-3′	5′-ATTYCACCGCTACACATG-3′
Bifidobacterium group	5′-GCGTGCTTAACACATGCAAGTC-3′	5′-CACCCGTTTCCAGGAGCTATT-3′
Escherichia coli	5′-AGAAGCTTGCTCTTTGCTGA-3′	5′-CTTTGGTCTTGCGACGTTAT-3′
Clostridium coccoides group	5′-AAATGACGGTACCTGACTAA-3′	5′-CTTTGAGTTTCATTCTTGCGAA-3′
Total bacteria (Universal primer)	5′-ACTCCTACGGGAGGCAGCAGT-3′	5′-ATTACCGCGGCTGCTGGC-3′

Table S3. Effects of *L. paracasei* HII01 on tissue TG accumulation in experimental groups.

Parameters (mg/g tissue)	NDC	ND-L	DMC	DM-L	DMM	DMM-L
Muscle TG	9.61 ± 0.20 [b]	10.10 ± 0.18 [b]	15.19 ± 0.80 [a]	9.66 ± 1.81 [b]	9.37 ± 0.98 [b]	8.11 ± 0.57 [b]
Hepatic TG	68.55 ± 4.52 [b]	72.07 ± 4.24 [b]	147.88 ± 18.02 [a]	115.86 ± 0.26 [c]	127.00 ± 18.93 [a]	131.68 ± 2.69 [a]

NDC, normal control rats; ND-L, normal control rats supplemented with *L. paracasei* HII01 (10^8 CFU/day), DMC, diabetic rats control; DM-L, diabetic rats supplemented with *L. paracasei* HII01 (10^8 CFU/day); DMM, diabetic rats treated with metformin 30 mg/kg; DMM-L, diabetic rats supplemented with combination of *L. paracasei* HII01 (10^8 CFU/day) and metformin 30 mg/kg; TG, triglyceride. All data are expressed as mean ± SEM. Different lowercase letters indicate significant differences among different groups ($p < 0.05$).

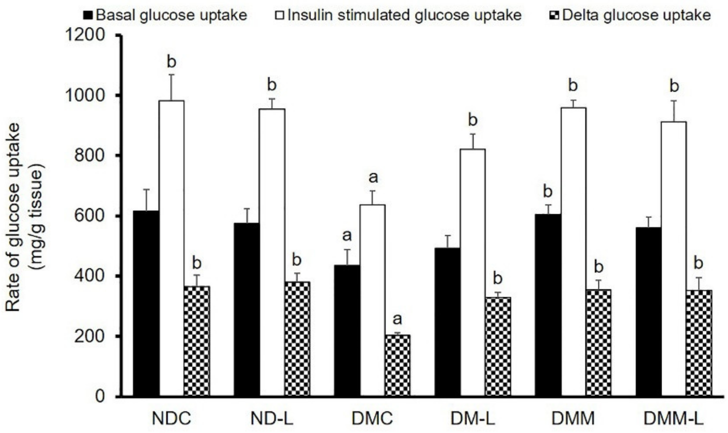

Figure S1. Effects of *L. paracasei* HII01 on *in vitro* glucose uptake in experimental groups. NDC, normal control rats; ND-L, normal control rats supplemented with *L. paracasei* HII01 (10^8 CFU/day), DMC, diabetic rats control; DM-L, diabetic rats supplemented with *L. paracasei* HII01 (10^8 CFU/day); DMM, diabetic rats treated with metformin 30 mg/kg; DMM-L, diabetic rats supplemented with combination of *L. paracasei* HII01 (10^8 CFU/day) and metformin 30 mg/kg. All data are expressed as mean ± SEM. Different lowercase letters indicate significant differences among different groups ($p < 0.05$).

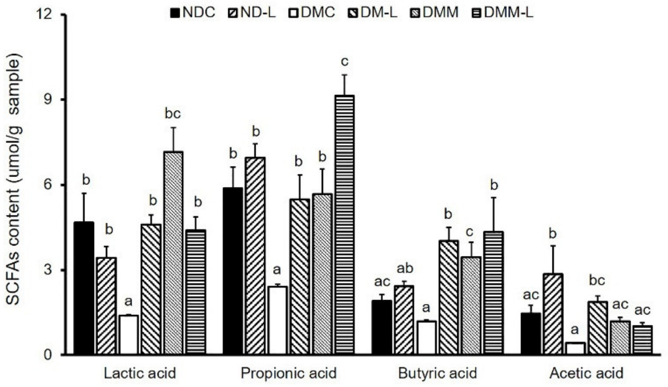

Figure S2. Effects of *L. paracasei* HII01 on cecal SCFAs of the experimental groups. NDC, normal control rats; ND-L, normal control rats supplemented with *L. paracasei* HII01 (10^8 CFU/day), DMC, diabetic rats control; DM-L, diabetic rats supplemented with *L. paracasei* HII01 (10^8 CFU/day); DMM, diabetic rats treated with metformin 30 mg/kg; DMM-L, diabetic rats supplemented with combination of *L. paracasei* HII01 (10^8 CFU/day) and metformin 30 mg/kg; SCFAs, short chain fatty acids. All data are expressed as mean ± SEM. Different lowercase letters indicate significant differences among different groups ($p < 0.05$).

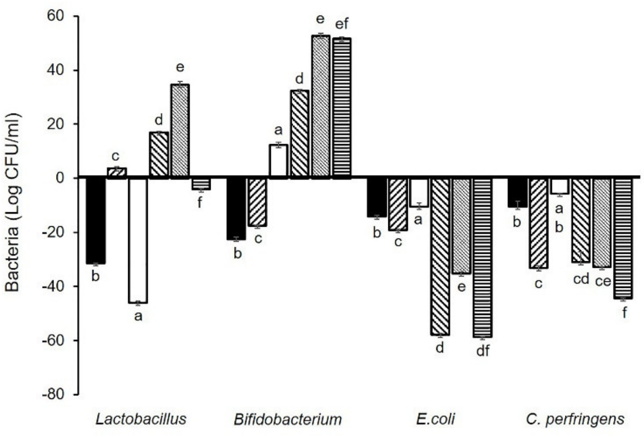

Figure S3. Effects of *L. paracasei* HII01 on the mean percent change in bacterial abundance from baseline analyzed in feces of the experimental groups. NDC, normal control rats; ND-L, normal control rats supplemented with *L. paracasei* HII01 (10^8 CFU/day), DMC, diabetic rats control; DM-L, diabetic rats supplemented with *L. paracasei* HII01 (10^8 CFU/day); DMM, diabetic rats treated with 30 mg/kg BW; DMM-L, diabetic rats supplemented with combination of *L. paracasei* HII01 (10^8 CFU/day) and metformin 30 mg/kg BW. All data are expressed as mean ± SEM. Different lowercase letters indicate significant differences among different groups ($p < 0.05$).
